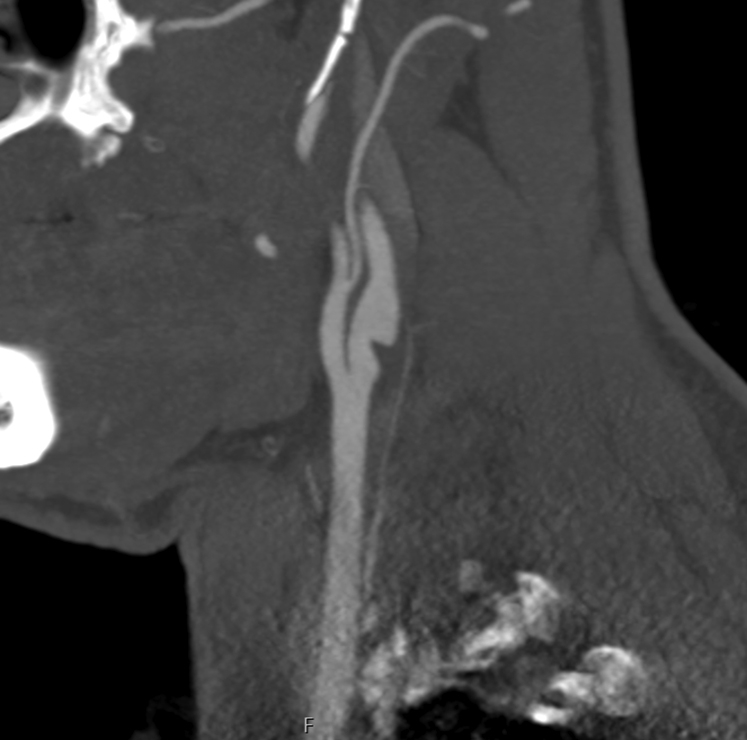# Large symptomatic carotid web in young African-Swedish man

**DOI:** 10.1016/j.jvscit.2021.11.009

**Published:** 2021-12-23

**Authors:** Peter Gillgren, Claes Skiöldebrand

**Affiliations:** Department of Clinical Science and Education, Karolinska Institutet, and Department of Surgery, Södersjukhuset, Stockholm, Sweden

**Keywords:** Carotid arteries, Carotid web, Cryptogenic stroke, Intimal fibromuscular dysplasia, Risk factors

Debate is ongoing regarding whether carotid web (CW)—a type of fibromuscular dysplasia of the intima—can cause brain embolization and, thereby, ischemic stroke. Sole medical treatment of the condition has been associated with high recurrence rates. Studies have shown a strong association with cryptogenic or occult ipsilateral ischemic stroke through embolization, with a high number of patients of African descendant included in reported case series.^1^, ^2^, ^3^

A 32-year-old nonsmoking Cameroon-born previously healty African-Swedish man, with no risk factors, had presented with a National Institutes of Health stroke scale score 11 stroke, with left-sided hemiplegia, aphasia, and central facial nerve palsy. Computed tomography (CT) angiography showed occlusions in M2, M3, and M4 and a 4 × 5-cm frontotemporal infarction of the right hemisphere. Duplex ultrasound and CT angiography revealed a carotid web in the right internal carotid artery (*A*/Cover). The left carotid was normal. The ensuing attempted intra-arterial thrombolysis showed that occluded right-sided M3 and M4 branches remained. Therefore, mechanical thrombectomy was performed with restoration of blood flow. The findings from transesophageal echocardiography, telemetry, and lipid and biomarkers of systemic inflammation measurements were all normal. After the stroke, the patient was discharged with dual antiplatelet treatment (aspirin and clopidogrel) for 3 weeks, followed by a single aspirin and atorvastatin at 40 mg. The patient provided written informed consent for the report of his case details and imaging studies (August 19, 2021).

Because the CW was considered the source of emboli and rehabilitation was successful (National Institutes of Health stroke scale score of 0 at 3 months), the patient was scheduled for delayed surgery and underwent carotid “webectomy.” The membranous structure removed was more prominent and voluminous than expected (*B* and *C*). Using a surgical dissector, the CW loosened easily (*D*). Because the artery was wide and the patient young, primary suture of the artery was performed. The patient was discharged with a prescription for aspirin for 4 months, and atorvastatin was discontinued after 1 month. His recovery was uneventful. Histologic examination showed fibromuscular dysplasia. The patient has been scheduled for a renal artery magnetic resonance imaging study.

The CW depicted in the CT in the present patient of African descent was considered the source of the ischemic stroke. CW can lead to embolic ischemic stroke. Thus, in countries with a low number of persons of African descent, CW could be underrecognized. After meticulous examination, these patients could be considered for carotid revascularization.

**Figure:**
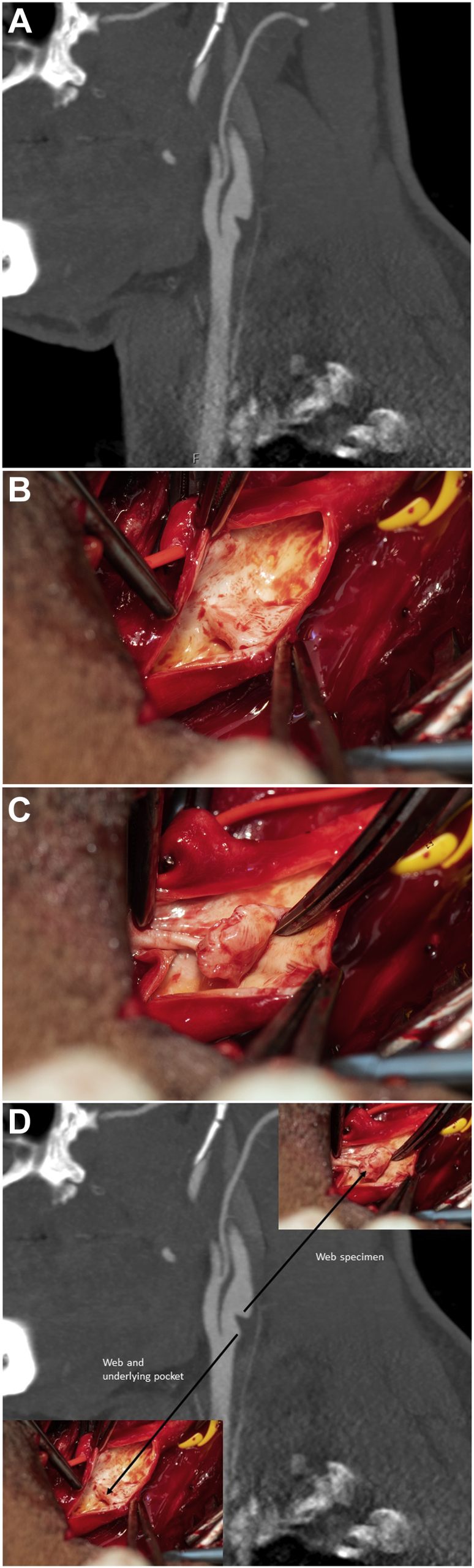

## Figures and Tables

**Cover Image:**